# Editorial: Current Trends of Insect Physiology and Population Dynamics: Modeling Insect Phenology, Demography, and Circadian Rhythms in Variable Environments

**DOI:** 10.3389/fphys.2018.00336

**Published:** 2018-04-05

**Authors:** Petros T. Damos, Sibylle C. Stoeckli, Alexandros Rigas

**Affiliations:** ^1^Pharmacy Department, University General Infectious Diseases Hospital of Thessaloniki AHEPA, Thessaloniki, Greece; ^2^Laboratory of Applied Zoology and Parasitology, Department of Crop Production, Field Crops and Ecology, Horticulture and Viticulture and Plant Protection, Faculty of Agriculture, Forestry and Natural Environment, Aristotle University of Thessaloniki, Thessaloniki, Greece; ^3^Research Institute of Organic Agriculture (FiBL), Frick, Switzerland; ^4^Department of Electrical and Computer Engineering, Democritus University of Thrace, Xanthi, Greece

**Keywords:** insects, population modeling, diapause, temperature, environment

Physiological processes of insects and related arthropods are strongly affected by abiotic factors in which temperature and photoperiod have a predominant function setting the limits of distribution in time and space. Other important factors, which may affect the development of insect populations, for a particular environment, may be related to alterations in humidity levels and type of available nutrition. In temperate climates, seasonal changes of the above environmental conditions may cause alterations in physiological processes which are further expressed at the macroscopic level through alterations in population dynamics and insect phenology.

Traditionally, such processes are described using certain type of models including empirical and conceptual modeling descriptions, which in their simplest forms are based on fundamental rules of temperature-dependent development. Recently, as we include more variables and proceed to more complex physiological systems make such models less accurate. As a result, several theoretical and experimental works have been carried to shade light on how physiology and population dynamics is affected by a particular environment. Additionally, when merged with current progresses in modeling and computation they offer new means in describing insect physiology and population dynamics.

In the present Research Topic in Frontiers in Invertebrate Physiology we embody a series of original works which highlight some of the current directions of this field. There is a great diversity of modeling studies included that goes across several insect taxa and ecosystems reflecting the magnitude of the effects of the physical environment on insect physiology and related population processes. However, most studies included take into account the impact of principal abiotic factors (temperature and photoperiod) as driving forces and use models (empirical, computational, conceptual and other) as a basic tool to describe and predict their effects on the physiological functioning at the macroscopic-population, or, cellular-individual levels.

One of the major challenges of modeling insect population dynamics is not only to include the effects of climates but also the importance of landscape complexity. Lux et al. are using the medfly (*Ceratitis capitata*) as a model organism, propose a “virtual farm” modeling concept for enhancement of site-specific pest management and validates its utility for Integrated Pest Management (IPM). In the same context, Lux in a method article, extends the modeling approach to gain insight into the environmental and landscape factors driving insect behavior and invasion dynamics and demonstrates *in silico* that incipient cryptic populations may occasionally occur in a territory despite that local climate and a fragmented landscape may be sub-optimal for their establishment.

Joshi et al. are using degree-day (DD) phenology models to predict the timing of the codling moth (*Cydia pomonella*) development and phenology events and illustrate the continuous need for validating the functionality of available degree-day models when applied under new environmental conditions. They also bring in to account the potential impact of changes in orchard environment and crop management practices on the biology and phenology of the species.

Another constraint in traditional degree-day models is that they ignore the life-history functions that are not primarily influenced by temperature, such as the transition between diapausing and non-diapausing life stages. In this direction, Nielsen et al. tackles the constrain of the utility of single parameters DD models and have developed a stochastic agent-based model for insects that overwinters as diapause adults and further describe the stage specific phenology and population dynamics of the insect species *Halyomorpha halys* across a variety of geographic regions. It is shown that agent-based models better approximate the phenology of a species that has strongly overlapping life stages and heterogeneity in its population traits.

Moreover, although a large body of literature is devoted to the development of temperature driven phenology models, yet the relevance of fine-scale climatic data and their effect on accurate modeling species population dynamics and its distribution remains a matter of debate. The study of Rebaudo et al. supports that in the absence of microclimate dataset, global climate models are best fitted to predict species abundances at large scales (and low settlement), while microclimate datasets best predict abundances at fine scales (and high resolution). Furthermore, Robinet et al. discuss how climate change could alter phenology in the pine processionary Moth, an emblematic species, and how climate spatial heterogeneity interacts with phenology, making the mechanism of range expansion more complex than initially thought.

Insect life cycle demography and related physiological process of constant climate may be likewise affected by the quality and quantity of nutrition, which is available as well as on other factors such as the presence of a light source. Thus, two papers deal with the effects other factors than temperature on the life cycle and certain demographic aspects of insects. In the first related study, Kouloussis et al. have developed a feeding quantification method for the medfly and have brought us a better understanding of the nutritional influences on medfly lifespan and its interplay with reproduction and provide certain parametric and non-parametric approaches. To determine the underlying mechanisms of insect phototactic behaviors Zhang et al. recorded the phototactic response of two morphs of the pea aphid *Acyrthosiphon pisum* in different nymphal instars to white. They have further analyzed the relationship between phototactic response and fecundity and conclude that that light signals are important for aphid dispersal and distribution, and are also essential for the pea aphids to cope with environmental changes.

The two following contributions deal with the outcome of specific external causes on the degree of different types of gene expression aspects. The first work of Kola et al. aimed to examine the effect of dsRNA molecules on silencing the genes cytochrome *CYP6* and aminopeptidase *APN* of the rice yellow stem borer (YSB), *Scirpophaga incertulas* by feeding larvae on dsRNA treated cut stems. It is indicated that larvae fed with dsRNA specific to the CYP6 and APN showed significant inhibition of maturation in a time dependent manner, a trend which may bear some interesting facets of pest management in affecting their populations. On the other hand, to develop more realistic predictions about the biological impact of climate change in biological invasions, Boher et al. have assessed the combined effects of the mean and the variance of temperature on the physiological tolerance in adults of the invasive fruit fly *Drosophila melanogaster* and the native *Drosophila gaucha*. Through the expression of heat shock protein (*hsp90*) as a biochemical response they suggest that historical biogeography may be an associated important feature of species under current and future variable climatic scenarios.

The two final review papers included focus on the description of insect circadian rhythms with emphasis to insect circadian clocks. The review of Ito and Tomioka provides a description of the heterogeneous nature of peripheral circadian clocks in the fruit fly *D. melanogaster* and their dependence on the central clock, and discuss their significance in the temporal organization of physiology in peripheral tissues/organs. They bring out new lines of evidence that clearly demonstrate that the oscillatory machinery and degree of independence from the central clock vary among the peripheral clocks and emphasize that we should pay attention to environmental cues other than light. Finally, Rivas et al. put emphasis on the molecular mechanisms and evolutionary dynamics of insect clocks in variable environments. Circadian rhythms may be expressed as oscillations in behavior, metabolism and physiology, that have a period close to 24 h and are controlled by an internal pacemaker that evolved under strong selective pressures imposed by environmental cyclical changes, mainly of light and temperature. They include additionally a conceptual model of the circadian clock in *Drosophila*, which depicts multiple interlocked loops that are affected by temperature and light/dark duration suggesting that light and temperature entrainment have a basic role.

Overall, the current Research Topic includes method articles, original research articles and reviews, which contribute toward a better understanding of modeling certain features of insect physiology at the population or individual level. Additionally, we feel that the current collection may be a motive for future research directions in studding insect physiology, demography and circadian rhythms in variable environments.

## Author contributions

PD proposed and organized the research topic. PD, SS, and AR, have made substantial, direct and intellectual contribution to the editorial, and approved it for publication.

### Conflict of interest statement

The authors declare that the research was conducted in the absence of any commercial or financial relationships that could be construed as a potential conflict of interest.

